# Comparative effectiveness of long-term acute care hospital versus skilled nursing facility transfer

**DOI:** 10.1186/s12913-020-05847-6

**Published:** 2020-11-11

**Authors:** Anil N. Makam, Oanh Kieu Nguyen, Michael E. Miller, Sachin J. Shah, Kandice A. Kapinos, Ethan A. Halm

**Affiliations:** 1 Department of Internal Medicine, UT Southwestern Medical Center, Dallas, TX USA; 2 Department of Population and Data Sciences, UT Southwestern Medical Center, Dallas, TX USA; 3 Division of Hospital Medicine, Chan Zuckerberg San Francisco General Hospital, San Francisco, USA; 4 Division of Hospital Medicine, University Hospital of UCSF, San Francisco, USA; 5 Division of Hospital Medicine, University of California San Francisco, San Francisco, USA; 6 RAND Corporation, Arlington, VA USA

**Keywords:** Post-acute care, Medicare, Older adults, Skilled nursing facility, Comparative effectiveness

## Abstract

**Background:**

Long-term acute care hospital (LTACH) use varies considerably across the U.S., which may reflect uncertainty about the effectiveness of LTACHs vs. skilled nursing facilities (SNF), the principal post-acute care alternative. Given that LTACHs provide more intensive care and thus receive over triple the reimbursement of SNFs for comparable diagnoses, we sought to compare outcomes and spending between LTACH versus SNF transfer.

**Methods:**

Using Medicare claims linked to electronic health record (EHR) data from six Texas Hospitals between 2009 and 2010, we conducted a retrospective cohort study of patients hospitalized on a medicine service in a high-LTACH use region and discharged to either an LTACH or SNF and followed for one year. The primary outcomes included mortality, 60-day recovery without inpatient care, days at home, and healthcare spending

**Results:**

Of 3503 patients, 18% were transferred to an LTACH. Patients transferred to LTACHs were younger (median 71 vs. 82 years), less likely to be female (50.5 vs 66.6%) and white (69.0 vs. 84.1%), but were sicker (24.3 vs. 14.2% for prolonged intensive care unit stay; median diagnosis resource intensity weight of 2.03 vs. 1.38). In unadjusted analyses, patients transferred to an LTACH vs. SNF were less likely to survive (59.1 vs. 65.0%) or recover (62.5 vs 66.0%), and spent fewer days at home (186 vs. 200). Adjusting for demographic and clinical confounders available in Medicare claims and EHR data, LTACH transfer was not significantly associated with differences in mortality (HR, 1.12, 95% CI, 0.94–1.33), recovery (SHR, 1.07, 0.93–1.23), and days spent at home (IRR, 0.96, 0.83–1.10), but was associated with greater Medicare spending ($16,689 for one year, 95% CI, $12,216–$21,162).

**Conclusion:**

LTACH transfer for Medicare beneficiaries is associated with similar clinical outcomes but with higher healthcare spending compared to SNF transfer. LTACH use should be reserved for patients who require complex inpatient care and cannot be cared for in SNFs.

**Supplementary Information:**

The online version contains supplementary material available at 10.1186/s12913-020-05847-6.

## Background

The use of post-acute care by hospitalized adults has increased by 50% over the past two decades [1, 2], and accounts for the single largest increase in Medicare spending [3]. Nearly half of hospitalized Medicare beneficiaries receive post-acute care at discharge [4], with increasing use with age [5]. Within the expanding post-acute care sector, long-term acute care hospitals (LTACHs) treat patients who require extended inpatient care. LTACHs were initially intended to care for those requiring prolonged mechanical ventilation, but the only official Medicare requirement for LTACH certification is to maintain an average length of stay of at least 25 days [6]. Consequently, LTACHs care for an expanded population with complex and prolonged illness, three-quarters of whom are not mechanically ventilated, but have a range of medical needs such as intravenous antibiotics, complex wound care, and dialysis [7–9]. As such, LTACHs are the most expensive post-acute care provider, and cost the Medicare program $4.5 billion annually [10].

Before the growth of LTACHs, the majority of hospitalized patients who were too sick to go home were instead discharged to skilled nursing facilities (SNFs), the principal post-acute care alternate [9, 11]. Due to overlap in levels of care with SNFs, there is considerable variation in LTACH use, with only half of the LTACH vs SNF transfer decision explained by differences in patients’ illness severity or complexity [7, 12]. Given that LTACHs are reimbursed at over three-fold higher rates than SNFs for comparable diagnoses, a head-to-head comparison is needed to examine the effectiveness of LTACHs.

For similarly sick older adults, LTACHs may improve outcomes by providing daily physician care, more favorable nurse-to-patient ratios, and more intensive interdisciplinary care such as complex wound care, speech therapy, and dietary assessments that may be unavailable in SNFs [13]. Alternately, LTACHs may lead to worse health outcomes as patients treated in LTACHs have higher rates of hospital-acquired infections than SNFs, many involving multidrug resistant pathogens associated with central line and urinary catheters, and mechanical ventilation [14–17]. Prior research using national Medicare data has shown that LTACH transfer is associated with better outcomes at lower costs for only the sickest of patients—those who have multiple organ failure for selected diagnoses and those with chronical critical illness requiring prolonged mechanical ventilation [18, 19]. However, these studies did not directly compare LTACH vs SNF transfer, as they included patients discharged elsewhere, including home. Additionally, these studies used Medicare administrative claims data for analyses, which lacks granular information on severity of illness.

Therefore, we conducted a comparative effectiveness study of LTACH versus SNF transfer for hospitalized adults using Medicare claims data linked to clinically detailed electronic health record (EHR) data, which is a novel approach to studying post-acute care outcomes. We hypothesized that after adjusting for severity of illness and physiologic disturbances based on clinical data uniquely available in the EHR, LTACHs will have comparable outcomes to patients transferred to SNFs, but incur greater healthcare spending.

## Methods

### Design, data sources, and study population

We conducted a retrospective cohort study comparing the effectiveness of LTACH vs SNF transfer using Medicare Research Identifiable File claims data (Denominator, MedPAR, Outpatient, Carrier, and Durable Medical Equipment files) linked to EHR data for adults admitted for any reason to an inpatient internal medicine service at one of six hospitals in north Texas between November 1, 2009 and October 30, 2010. The six hospitals are part of two health systems: Parkland Health & Hospital System (one large urban, public safety-net hospital) and Texas Health Resources (five community, nonprofit hospitals). All six hospitals used Epic EHR (Epic Systems Corporation, Verona, WI). Details of this EHR cohort have been published [20, 21]. The Dallas-Fort Worth metroplex has among the highest LTACH use nationwide, thus making this particular healthcare market well-suited to study the effectiveness of LTACHs [7, 8].

Among this EHR cohort, we included Medicare fee-for-service beneficiaries who were subsequently transferred to an LTACH or SNF on the same or next day after hospital discharge using a temporally adjacent claims algorithm applied to the MedPAR files [22]. We identified LTACHs and SNFs by the Centers for Medicare and Medicaid Services (CMS) provider number, which are based on Medicare certification. We confirmed LTACHs by review of the facility name followed by an Internet search if the facility type was uncertain. To enable a lookback to ascertain utilization and comorbidities prior to the index episode of care, we excluded patients without fee-for-service Medicare (Parts A or B), or those with Medicare Advantage (Part C) at any time in the prior 6 months. We excluded patients with an acute care hospitalization of greater than 20 days since these patients were excluded from the original EHR cohort, and thus were missing clinical data at discharge. If patients had multiple hospitalizations leading to LTACH or SNF transfer during our study period, we selected the first one as the index episode of care. All eligible patients were included regardless of prior use of hospice or long-term care. We used Medicare claims data to follow patients for one year after the transfer date to assess outcomes, unless censored for death, loss of Medicare fee-for-service insurance, or gain of Medicare Advantage.

### Outcomes

Our primary clinical outcomes included all-cause mortality, recovery, and days spent at home. We ascertained vital status and dates of death from Medicare Denominator files. We defined recovery as achieving 60 consecutive days without care in a hospital (inpatient or observation stay) or post-acute care facility (LTACH, SNF or inpatient rehabilitation facility [IRF]). Sixty days without inpatient care is also the time period CMS uses to define the end of one benefit period (a spell of illness) and the start of a new benefit period. Days spent at home was defined as the days during the study period that were not spent in a hospital or an inpatient post-acute care facility, and included time spent on home hospice. Days at home is a patient-centered outcome, and is associated with better self-rated health and functioning [23–25].

We ascertained spending outcomes from Medicare’s perspective as well as from societal perspective (total spending from all payers, including Medicare, supplemental insurance and out-of-pocket expenses). For both Medicare and all payer perspectives, we examined spending for three different time periods: (1) for the index episode of care, which included the acute care hospitalization plus either the initial LTACH or SNF stay; (2) for the post-index episode of care, which included spending for any subsequent hospitalizations and inpatient post-acute care stays (LTACH, SNF, IRF) after the index LTACH or SNF stay, but within 1-year of the initial hospital admission; and (3) total 1-year spending from the time of hospitalization.

### Covariates

We included covariates available in either Medicare data or EHR data that were related to the LTACH and SNF transfer decision and to clinical and spending outcomes based on prior literature and our group’s multidisciplinary expertise [7, 18, 19]. From Medicare claims data, we included age, sex, race/ethnicity, prior healthcare utilization, prior durable medical equipment use (wheelchair, home hospital bed, oxygen), Charlson Comorbidity Index, hospital and intensive care unit length of stay, primary diagnosis (major comorbidity or complication designation, type, and diagnosis resource group resource intensity weight), selected secondary diagnoses, and selected intensive treatments or procedures (mechanical ventilation, tracheostomy, total parenteral nutrition, permanent feeding tube, central venous line, dialysis, and excisional wound debridement) (see Table 1 for details).

**Table 1:** Baseline Characteristics

	SNF (***n*** = 2865)	LTACH (***n*** = 638)	p
**Characteristics from Medicare Claims Data**			
Age, years, median (IQR)	82 (74–88)	71 (63–79)	<.001
Female gender, n (%)	1907 (66.6)	322 (50.5)	<.001
Non-Hispanic White	2408 (84.1)	440 (69.0)	<.001
Prior hospitalization ≥2, n (%)	152 (5.3)	54 (8.5)	.001
Prior LTAC stay, n (%)	105 (3.7)	80 (12.5)	<.001
Prior SNF stay, n (%)	569 (19.9)	111 (17.4)	.16
Wheelchair use, n (%)	337 (11.8)	118 (18.5)	<.001
Home hospital bed, n (%)	138 (4.8)	50 (7.8)	.002
Oxygen, n (%)	406 (14.2)	155 (24.3)	<.001
Charlson Comorbidity Index, median (IQR)	0 (0–2)	1 (0–3)	<.001
Length of stay of hospitalization, days, median (IQR)	6 (4–9)	10 (7–14)	<.001
ICU stay ≥3 days during hospitalization, n (%)	253 (8.8)	155 (24.3)	<.001
Primary diagnosis			
DRG resource intensity weight, median (IQR)	1.38 (0.95–1.84)	2.03 (1.44–3.29)	<.001
DRG with a MCC designation, n (%)	1224 (42.7)	368 (57.7)	<.001
Respiratory Major Diagnostic Category, n (%)	345 (12.0)	103 (16.1)	.005
Circulatory Major Diagnostic Category, n (%)	492 (17.2)	113 (17.7)	.75
Musculoskeletal Major Diagnostic Category, n (%)	562 (19.6)	100 (15.7)	.02
Secondary diagnoses, n (%)			
Respiratory failure	288 (10.1)	161 (25.2)	<.001
Sepsis	178 (6.2)	103 (16.1)	<.001
Diabetes mellitus	215 (7.5)	90 (14.1)	<.001
Skin, soft-tissue, or joint infection	114 (4.0)	181 (28.4)	<.001
Chronic skin ulcer	201 (7.0)	131 (20.5)	<.001
Device, graft, or implant complication	67 (2.3)	45 (7.1)	<.001
Complication of care	130 (4.5)	56 (8.8)	<.001
Delirium or dementia	672 (23.5)	46 (7.2)	<.001
Selected intensive treatments or procedures, n (%)			
Mechanical ventilation			<.001
Transient (< 96 h)	185 (6.5)	81 (12.7)	
Prolonged (≥96 h)	16 (0.6)	53 (8.3)	
Tracheostomy	20 (0.7)	54 (8.5)	<.001
Total parenteral nutrition	25 (0.9)	20 (3.1)	<.001
Permanent feeding tube	170 (5.9)	87 (13.6)	<.001
Central venous line	801 (28.0)	429 (67.2)	<.001
Dialysis	163 (5.7)	130 (20.4)	<.001
Excisional debridement	104 (3.6)	127 (19.9)	<.001
**Characteristics from EHR Data**			
Married, n (%)	820 (28.6)	231 (36.2)	<.001
Median income per ZIP code of residence, $1000, med (IQR)	53.5 (42.7–67.3)	49.5 (39.2–64.8)	<.001
Transferred directly from the ICU, n (%)	66 (2.3)	87 (13.6)	<.001
Laboratory values at discharge, median (IQR)			
Albumin, g/dL	2.9 (2.5–3.3)	2.6 (2.1–3.0)	<.001
Hematocrit, %	32.3 (29.2–35.7)	30.6 (27.9–33.7)	<.001
Blood urea nitrogen, mg/dL	20 (14–29)	23 (15–38)	<.001
Creatinine, mg/dL	0.86 (0.70–1.20)	0.99 (0.70–1.70)	<.001
Sodium, mEq/L	138 (136–141)	138 (135–141)	0.74
Platelet count, cells/μL	220 (170–286)	249 (184–326)	<.001
White blood cell count, cells/μL	8.3 (6.5–10.4)	9.2 (7.2–11.8)	<.001
Vital Signs at discharge, median (IQR)			
Pulse, beats/minute	81 (72–91)	85 (74–97)	<.001
Systolic blood pressure, mm Hg	121 (109–134)	117 (103–132)	<.001
Respiratory rate, breaths/minute	20 (18–20)	20 (18–22)	<.001
Temperature, °F	98.1 (97.6–98.6)	98.2 (97.6–98.7)	.046
Oxygen saturation, %	95 (94–97)	96 (93–97)	0.86
Pain score at discharge, median (IQR)	0 (0–5)	0 (0–6)	<.001
Noninvasive positive pressure ventilation on discharge, n %	56 (2.0)	37 (5.8)	<.001
Intravenous diuretics on day of discharge, n (%)	25 (0.9)	24 (3.8)	<.001
Abbreviations: ICU, intensive care unit; DRG, diagnosis related group			

From the EHR data, we included additional socioeconomic information (marital status and proxy of income) and clinically detailed data on severity of illness at the time of discharge that was unavailable in the Medicare claims data (unit at time of transfer, laboratory test values, vital signs, pain score, use of noninvasive positive pressure ventilation, and use of intravenous diuretics).

### Subcohort

To examine the policy relevance and robustness of our findings, we developed a subgroup of patients who were most representative of the contemporary LTACH population and retained clinical equipoise between LTACH and SNF transfer. Beginning in fiscal year 2020, the CMS site-neutral payment policy will substantially decrease reimbursement for LTACH admissions among patients who did not have an intensive care unit stay of at least 3 days during the preceding acute care hospitalization or did not require prolonged mechanical ventilation (≥4 days). For this subcohort, we excluded patients who did not meet these criteria, since LTACH transfer for these patients currently would be less likely given the decreased financial incentives. Given the concern that SNFs lacked the experience and expertise to care for patients requiring mechanical ventilation, we also excluded patients who required mechanical ventilation after transfer.

### Statistical analyses

We calculated descriptive statistics to compare characteristics of patients transferred to an LTACH versus SNF. For comparative effectiveness analyses, we conducted three sequential regression models for each outcome to compare differences between patients transferred to an LTACH vs a SNF: 1) a univariate unadjusted model; 2) a multivariable model adjusting for confounders available from Medicare claims data, which has been the sole dataset used in this literature; and 3) a multivariable model adjusting for confounders available in both Medicare and EHR data, the latter of which is a novel approach to study the comparative effectiveness of LTACHs. This sequential modeling approach allowed us to estimate the independent association of LTACH transfer (vs SNF) on outcomes, and explore the impact of including additional measures of severity of illness and physiologic reserve that are unavailable in administrative claims data. For each model, we computed the relative effect size and the absolute difference estimated from marginal effects post-model estimation.

For Medicare data we had complete data for all covariates and outcomes. For EHR data, we were missing data for 0.1% of vital signs and up to 2.7% for laboratory test values, except for albumin (19.5% missing), aspartate aminotransferase (AST, 23.9%), and total bilirubin (23.8%) (see Appendix Table S1 for patterns of missing data). We assumed the missing data to be missing at random based on patterns of known covariates, and used multiple imputation using chained equations with 10 imputations. Diagnostic tests confirmed adequate convergence and ensured in-range values. All analyses were conducted on each imputed data set, and results were combined into a single estimate using the standard combining procedures.

For mortality, we used time-to-event Kaplan-Meier to examine survival curves, and Cox proportional hazard models to estimate the hazard ratio (HR) of death. The proportional hazards assumption was tested using Schoenfeld residuals and time-dependent covariates without any evidence of violations. To estimate the subdistribution hazard ratio (SHR) of achieving a 60-day recovery for patients transferred to an LTACH, we used Fine and Gray cumulative incidence function curves accounting for competing risks of hospice enrollment or death. To estimate the incidence rate ratio (IRR) for days spent at home after LTACH transfer, we used negative binomial regression with follow-up time before censoring as the exposure variable.

For spending analyses for the index episode of care and for total one-year follow-up, we used gamma generalized linear regression models with an identify link function. Since a proportion of patients did not incur any subsequent spending after the initial LTACH or SNF transfer, to estimate spending for the post-index episode of care, we constructed a two-part model. The first part of the model predicted whether spending for a patient was greater than zero. The second part of the model, conditional on positive spending, estimated spending using a gamma generalized linear regression model with log link function. All analyses were conducted using Stata 12.0.

## Results

### Description of cohort

Of 5603 potentially eligible hospitalizations to an internal medicine service at one of six hospitals in north Texas leading to an LTACH or SNF transfer, we included 3503 index episodes of care among unique Medicare beneficiaries (see Appendix Table S2).

From the Medicare data, patients transferred to LTACHs compared to SNFs were younger (median age of 71 vs. 82); less likely to be a woman (50.5% vs. 66.6%) or non-Hispanic white (69.0% vs. 84.1%); more likely to use durable medical equipment prior to admission (18.5% vs. 11.8% for wheelchair; 7.8% vs 4.8% for home hospital bed; and 24.3% vs 14.2% for home oxygen), have a greater comorbidity burden (median Charlson Comorbidity Index of 1 vs. 0), have longer hospital length of stay prior to transfer (median 10 vs. 6 days), have a prolonged intensive care unit (ICU) stay (24.3% vs. 8.8%) and have severe, complex and resource intensive diagnoses (median diagnosis related group weight of 2.03 vs 1.38, and greater incidence of secondary diagnoses), and undergo more intensive procedures (8.3% vs. 0.6% for prolonged mechanical ventilation, 8.5% vs. 0.7% for tracheostomy, 13.6% vs. 5.9% for permanent feeding tube; 67.2% vs. 28.0% for central venous line, 20.4% vs. 5.7% for dialysis, and 19.9% vs 3.6% for excisional wound debridement) (**see** Table 1, *p* < 0.001 for all analyses specified).

From the EHR data, patients transferred to LTACH were more likely to be married (36.2% vs. 28.6%), be transferred directly from the ICU (13.6% vs 2.3%), have lower albumin (median, 2.6 vs. 2.9 g/dL) and hematocrit (median, 30.6 vs. 32.3%), greater BUN (median, 23 vs. 20 mg/dL), creatinine (median, 0.99 vs. 0.86 mg/dL), pulse (median, 85 vs. 81 beats/minute), and use of noninvasive positive pressure ventilation at (5.8% vs 2.0%), but clinically similar blood pressure, respiratory rate, temperature, oxygen saturation, and pain score at the time of discharge (*p* < 0.001 for all analyses where differences specified).

Patients transferred to an LTACH spent a median of 25 days (interquartile range, IQR, 16–33) in the LTACH and patients transferred to a SNF spent a median of 24 days (IQR, 12–50) in the SNF.

### Clinical outcomes

#### Mortality

**Fig. 1:**
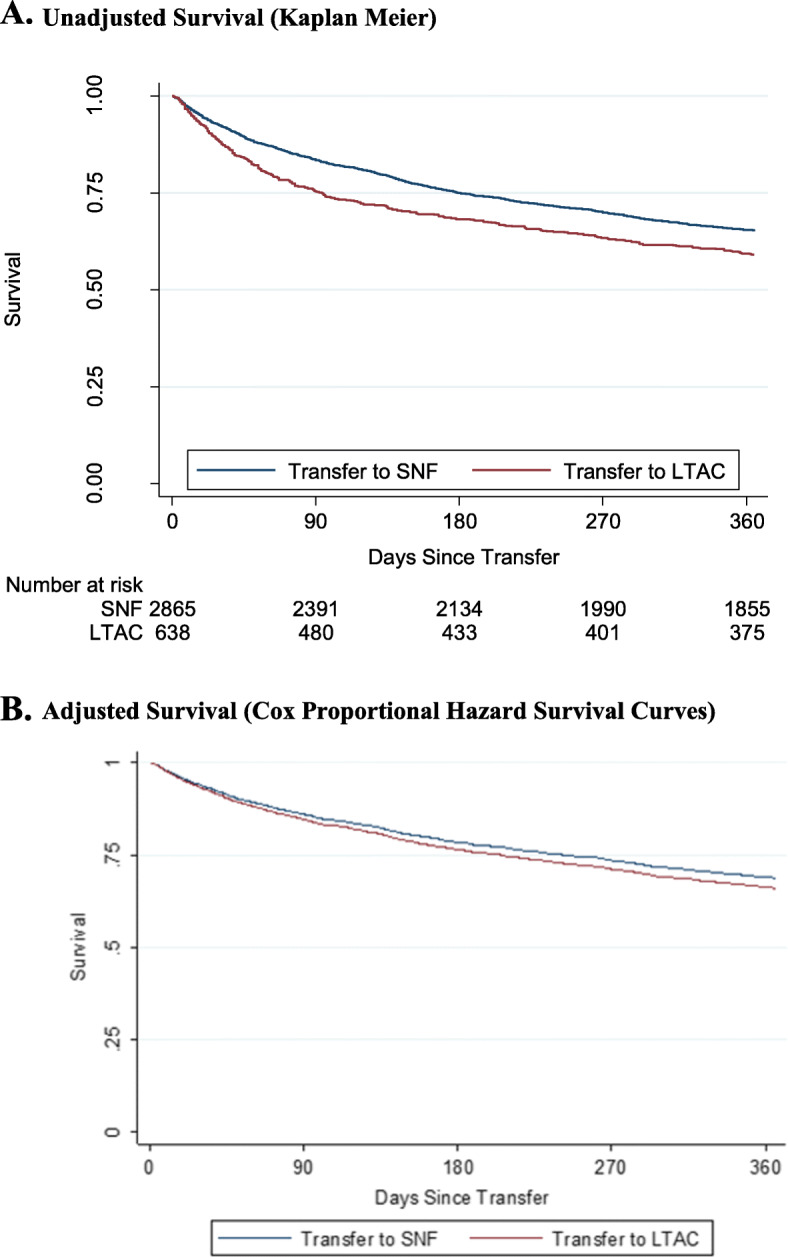
Mortality after LTACH vs. SNF Transfer Among Hospitalized Adults. **a** Unadjusted Survival (Kaplan Meier). **b** Adjusted Survival (Cox Proportional Hazard Survival Curves)

In unadjusted analyses, more patients transferred to an LTACH died (Fig. 1**a**, Table 2). At 1 year post transfer, unadjusted mortality was 40.9% vs. 35.0% for LTACH vs SNF patients respectively (*p* < .001). Adjustment for confounders available in Medicare data attenuated the HR from 1.25 (95% CI, 1.08–1.43) to 1.18 (95% CI, 1.00–1.39). The association was further attenuated with inclusion of additional confounders from the EHR data, and was no longer statistically significant (HR, 1.12; 95% CI, 0.94–1.33), with 3-month and 1-year adjusted mortality of 82.5% vs 84.0 and 58.5% vs 61.2% for LTACH vs SNF patients respectively (Fig. 1b).

**Table 2:** Clinical Outcomes Associated with LTACH vs. SNF Transfer Among Hospitalized Adults (*n* = 3503)

	All-Cause Mortality, HR	60-Day Recovery, SHR	Days at Home, IRR
Unadjusted	1.25 (1.08–1.43)	0.92 (0.82–1.02)	0.93 (0.83–1.04)
Adjusted using Claims Data	1.18 (1.00–1.39)	0.98 (0.86–1.13)	0.96 (0.83–1.10)
Adjusted using Claims + EHR Data	1.12 (0.94–1.33)	1.07 (0.93–1.23)	0.96 (0.83–1.10)
Abbreviation: LTACH, long-term acute care hospital; SNF, skilled nursing facility; HR, hazard ratio; SHR, subhazard ratio; IRR, incidence rate ratio			

#### Recovery

In the unadjusted analysis, fewer patients transferred to an LTACH recovered (SHR, 0.92; 95% CI, 0.82–1.02), but this was not statistically significant. The unadjusted absolute probability for achieving a 60-day recovery three months after transfer was 22.6% for LTACH patients versus 24.5% for SNF patients. At one year, the unadjusted probability of recovery was 62.5% for LTACH vs 66.0% for SNF patients. Adjustment for confounders from Medicare data attenuated the association (Table 2). After further inclusion of EHR data, the association changed directions but was statistically insignificant (SHR 1.07, 95% CI, 0.86–1.13).

#### Days at home

In the unadjusted analysis, patients transferred to an LTACH versus a SNF spent a similar number of days at home (IRR, 0.93, 95% CI, 0.83–1.10; difference of − 14 days, 95% -35-8). After adjustment using Medicare data, this non-significant association was attenuated even further to the null (Table 2, Appendix Table S3). Further adjustment with EHR data did not affect the estimate or precision.

### Health care spending

#### Medicare spending

In unadjusted analyses, patients transferred to LTACHs (vs SNFs) incurred considerably more Medicare spending for all time periods (the index of episode difference of $30,358; post-index episode of care difference of $10,881; total 1-year difference of $41,309, *p* < 0.001 for all; Table 3). After adjustment using Medicare data, differences for the index episode of care ($20,443) and total one-year spending ($16,294) were lower, but still financially and statistically significant. Further adjustment using EHR data did not meaningful change estimates for spending for any of the three time periods.

**Table 3:** Healthcare Spending after LTACH vs. SNF Transfer among Hospitalized Adults

	LTACH, $	SNF, $	Difference, $ (95% CI)	***P***-value
**Medicare Spending**				
**Index episode of care**^**a, b**^				
Unadjusted	51,822	21,465	30,358 (27,985-32,730)	<.001
Medicare model	43,630	23,186	20,443 (18,511-22,375)	<.001
Medicare & EHR model	43,721	23,179	20,542 (18,603-22,481)	<.001
**Post-Index episode of care**^**c,d**^				
Unadjusted	32,724	21,843	10,881 (7325-14,437)	<.001
Medicare model	22,788	24,121	-1334 (− 4477-1810)	0.41
Medicare & EHR model	22,790	24,137	-1347 (− 4518-1825)	0.41
**Total 1-year spending**^**b**^				
Unadjusted	84,652	43,343	41,309 (36,304-46,313)	<.001
Medicare model	64,141	47,847	16,294 (11,859-20,728)	<.001
Medicare & EHR model	64,476	47,787	16,689 (12,216-21,162)	<.001
**All Payer Spending**				
**Index episode of care**^**a,b**^				
Unadjusted	54,557	24,705	29,851 (27,267-32,435)	<.001
Medicare model	45,793	26,561	19,232 (16,966-21,498)	<.001
Medicare & EHR model	45,959	26,546	19,414 (17,137-21,690)	<.001
**Post-index episode of care**^**c,d**^				
Unadjusted	37,611	24,804	12,807 (8728-16,887)	<.001
Medicare model	26,336	27,333	− 997 (− 4623-2629)	0.59
Medicare & EHR model	26,348	27,338	− 991 (− 4656-2675)	0.60
**Total 1-year spending**^**b**^				
Unadjusted	92,310	49,541	42,769 (37,346-48,191)	<.001
Medicare model	69,798	54,461	15,337 (10,452-20,222)	<.001
Medicare & EHR model	70,193	54,399	15,795 (10,858-20,731)	<.001

*Abbreviations: LTACH* long-term acute care hospital, *SNF* skilled nursing facility

^a^ Includes the initial acute care hospitalization and subsequent LTACH or SNF stay

^b^ We computed marginal effects after gamma generalized linear regression model estimation with an identity link function to generate predicted spending for LTACH and SNF cohorts, and contrasts for the difference and robust standard errors using the delta method

^c^ Includes all hospitalizations and inpatient post-acute care stays (LTACH, SNF, IRF) after the index LTACH or SNF stay through 1-year after the date of transfer

^d^ We computed marginal effects after a two-part model, and contrasts for the difference and robust standard errors using the delta method. The first part of the model predicted whether spending for a patient was greater than zero. The second part, conditional on positive spending, predicted spending for LTACH and SNF patients using a gamma generalized linear regression model with a log link function

#### All payer spending

Our analyses for all payer spending revealed similar differences between LTACH and SNF transfer, as well as similar patterns after adjustment for Medicare and EHR data as our analyses of Medicare spending (Table 3). The estimated absolute spending for LTACH vs SNF transfer was higher for each time period, reflecting the inclusion of additional sources of spending beyond Medicare. In our fully adjusted model using both Medicare and EHR data, transfer to LTACH was associated with an additional total one-year spending of $15,795 (95% CI, $10,858-20,731).

### Subcohort analysis

Among our subcohort of 1055 patients who would have been exempt from reduced reimbursement in the current site-neutral payment era, but did not require prolonged mechanical ventilation, our findings for all clinical and spending outcomes were similar to analyses for our overall cohort (Appendix Tables S4 and S5).

## Discussion

In this retrospective post-acute care cohort study linking Medicare claims to clinically detailed EHR data, we found that LTACH transfer for Medicare beneficiaries hospitalized to an inpatient medicine service in a high-LTACH use region was associated with similar clinical outcomes as SNF transfer (mortality, 60-day recovery, and days spent at home), but with greater healthcare spending (approximately $16,000 per transfer). When we focused on the subgroup of patients most representative of the contemporary LTACH population (i.e. meet site-neural payment criteria for full reimbursement), but did not receive mechanical ventilation after transfer, since these patients may not have been able to be cared for in SNFs, our findings remained consistent with our overall analyses. As payers and health systems are increasingly focused on maximizing value, limiting LTACH transfers for patients who truly need prolonged hospital-level care beyond the capabilities of SNFs, could result in comparable clinical outcomes at a much lower cost. This is especially pertinent during the current pandemic, as LTACHs may play an outsized role in managing the projected surges of patients with prolonged respiratory failure from COVID− 19 [26, 27].

Our findings complement previous studies on the effectiveness of LTACHs which used different comparison groups, datasets, and analytic approaches [9, 11, 18, 19]. Our head-to-head comparison with SNFs, which is the principal post-acute care alternate to LTACHs, found similar effectiveness for LTACH transfer as studies that compared LTACH transfer to patients discharged to SNFs, IRFs, or home. Due to our limited sample size, we were unable to explore whether there were subgroups of patients transferred to LTACHs that would benefit compared to SNFs as previous studies have found, most notably chronically critically ill patients and those with multiple organ failure [18, 19]. We also found that adjustment for confounders available in EHR data, which included more information on clinical severity and complexity than Medicare administrative claims data, were necessary for examining mortality and recovery to better account for selection bias between patients transferred to LTACHs versus SNFs. However, the inclusion of EHR data did not meaningful change our findings for days at home or healthcare spending, which suggests that Medicare data is adequate for risk adjustment for these outcomes, and potentially even for public reporting purposes or pay-for-performance designs. Lastly, our sequential regression modeling approach approximated findings from studies that employed instrumental variable analytic techniques. In the absence of a randomized controlled trial of which post-acute care setting optimizes outcomes, it is reassuring that different approaches yield similar conclusions regarding the overall effectiveness of LTACH transfer.

While SNF transfer in theory may lead to comparable clinical outcomes, in practice, SNFs may lack the expertise, experience, and resources to provide care for this population with complex and serious illness [28]. This is likely most pertinent in regions with high LTACH use, such as the South and Ohio Valley, than in the Pacific Northwest, North, and New England regions where LTACH use is scarce [7, 8, 12]. In regions with high LTACH use, SNFs may not have developed or sustained the capabilities to adequately care for this population. Two recent federal policies have changed the financial incentives to shift less medically complex patients from LTACHs to SNFs. Beginning October 2020, the CMS site-neutral payment policy will considerably decrease the reimbursement to LTACHs for patients who did not have a qualifying ICU stay of 3 or more days prior to transfer or did not receive prolonged mechanical ventilation in the LTACH [29]. Effective beginning October 2019, the CMS Patient-Driven Payment Model (PDPM) will increase the SNF per-diem reimbursement by 10–30% for patients who require non-rehabilitation care, such as antibiotic infusions, dialysis, wound care, and ventilator support [30, 31].

Our study should be interpreted in the context of certain limitations. First, our findings may not generalize to low LTACH use regions where the patients cared for in LTACHs are sicker than those in high-use regions, such as the Dallas-Fort Worth metroplex [29]. Second, due to inclusion criteria of the original EHR cohort that we used for this study, we were only able to study patients admitted to an internal medicine service, so our findings may not apply to those with trauma, surgical, or neurologic conditions. Third, we were only able to compare outcomes available in Medicare data, so we do not know whether the greater intensity of care provided in LTACHs may have resulted in better cognitive or functional recovery or quality of life. However, patients had similar 60-day recovery and time spent at home, which may be surrogate measures of these more patient-oriented outcomes. Fourth, our analyses provide an estimate of the average treatment effect of LTACH versus SNF transfer, and does not account for variability of care within each post-acute care setting that may affect outcomes. Fifth, our analyses may not fully account for the differences between LTACH and SNF patients. However, we included detailed severity of illness measures available in both the Medicare claims and EHR data, including proxies of disability and frailty.

## Conclusions

In this novel post-acute care cohort where we linked Medicare claims to clinically detailed EHR data, we found that LTACH transfer for Medicare beneficiaries hospitalized to an internal medicine service in a high-LTACH use region was not associated with improved clinical outcomes but associated with considerably greater healthcare spending. Our findings support federal policies to shift patients from the more intensive LTACHs to SNFs by decreasing reimbursement for less sick patients in LTACHs and increasing reimbursement for more complex patients in SNFs.

## Supplementary Information

**Additional file 1 Appendix Table S1**. Missing EHR Data (n=3,503). **Appendix Table S2**. Study Flow Table. **Appendix Table S3**. Adjusted Number of Days Spent at Home after Transfer. **Appendix Table S4**. Sensitivity Analysis of Clinical Outcomes. **Appendix Table S5**. Sensitivity Analysis for Spending.

## Data Availability

The datasets generated and analyzed during the current study are not publicly available due to a restricted data use agreement executed between the University of Texas Southwestern and CMS.

## Abbreviations

LTACH: Long-term acute care hospital
SNF: Skilled nursing facility
EHR: Electronic health records
CMS: Centers for Medicare and Medicaid Services
IRF: Inpatient rehabilitation facility
HR: Hazard ratio
SHR: Subdistribution hazard ratio
IRR: Incidence rate ratio
ICU: Intensive care unit
