# Supplementary material for: Comparative effectiveness of long-term acute care hospital versus skilled nursing facility transfer
Source: BMC Health Serv Res. 2020 Nov 11;20:1032. doi: 10.1186/s12913-020-05847-6 (PMC7656509; doi:10.1186/s12913-020-05847-6)
Supplement: Supplementary file 1 — Additional file 1 Appendix Table S1. Missing EHR Data (n=3,503). Appendix Table S2. Study Flow Table. Appendix Table S3. Adjusted Number of Days Spent at Home after Transfer. Appendix Table S4. Sensitivity Analysis of Clinical Outcomes. Appendix Table S5. Sensitivity Analysis for Spending. [file 12913_2020_5847_MOESM1_ESM.docx]

**SUPPLEMENTARY APPENDIX**

**Comparative Effectiveness of Long-Term Acute Care Hospital versus Skilled Nursing Facility Transfer**

| Appendix Table S1. Missing EHR Data (n=3,503)…….……….......................................... | 2 |
| --- | --- |
| Appendix Table S2. Study Flow Table…………………………………………………….. | 3 |
| Appendix Table S3. Adjusted Number of Days Spent at Home after Transfer…………… | 4 |
| Appendix Table S4. Sensitivity Analysis of Clinical Outcomes…………………………... | 5 |
| Appendix Table S5. Sensitivity Analysis for Spending…………………………………… | 6 |
|  |  |

| **Appendix Table S1.** Missing EHR Data (n=3,503) | |
| --- | --- |
| **EHR Covariates** | **Missing value, n (%)** |
| Albumin | 684 (19.5%) |
| Hematocrit | 71 (2.0%) |
| Blood urea nitrogen (BUN) | 45 (1.3%) |
| Creatinine | 45 (1.3%) |
| Aspartate aminotransferase (AST) | 837 (23.9%) |
| Total bilirubin | 835 (23.8%) |
| Sodium | 45 (1.3%) |
| Potassium | 43 (1.2%) |
| Platelets | 91 (2.6%) |
| White blood cells (WBC) | 93 (2.7%) |
| Pulse | 5 (0.1%) |
| Systolic blood pressure | 5 (0.1%) |
| Respiratory rate | 5 (0.1%) |
| Temperature | 5 (0.1%) |
| Oxygen saturation | 37 (1.1%) |
| Pain | 11 (0.3%) |

| **Appendix Table S2.** Study Flow Table | | |
| --- | --- | --- |
| **Exclusion Criteria** | **Excluded, n (%)** | **Included, n** |
| Hospitalized Medicare beneficiaries transferred to an LTACH or SNF ^a^ | - | 5,603 |
| Does not have Parts A and B, or has Part C for ≥1 month during 6 month lookback | 528 (10.4%) | 4,535 |
| Not first eligible index episode of care in study period | 802 (17.7%) | 3,733 |
| Hospital length of stay > 20 days (data unavailable in EHR dataset) | 222 (5.9%) | 3,511 |
| Duplicate patients | 8 (0.2%) | 3,503 |
| Abbreviations: LTACH, long-term acute care hospital; SNF, skilled nursing facility; EHR, electronic health record  ^a^ patients admitted for any reason to an internal medicine service at one of six hospitals in north Texas | | |

| **Appendix Table S3.** Adjusted Number of Days Spent at Home after LTACH vs. SNF Transfer (n=3,503) | | | | |
| --- | --- | --- | --- | --- |
|  | **LTACH** | **SNF** | **Difference** |  |
| **Model** | Adjusted Number of Days at Home (95% CI) | | | P-value |
| Unadjusted | 186 (167-205) | 200 (190-209) | -14 (-35 to +8) | 0.22 |
| Claims Data Model | 190 (167-213) | 199 (189-209) | -9 (-35 to +18) | 0.51 |
| Claims & EHR Data Model | 191 (167-215) | 199 (189-210) | -8 (-35 to +19) | 0.54 |
| Abbreviations: LTACH, long-term acute care hospital; SNF, skilled nursing facility | | | | |

| **Appendix Table S4.** Sensitivity Analysis of Clinical Outcomes Associated with LTACH vs. SNF Transfer Among Hospitalized Adults Exempt from Site-Neutral Payment and Without Mechanical Ventilation (n=1,055) ^a^ | | | |
| --- | --- | --- | --- |
|  | **All-Cause Mortality, HR** | **60-Day Recovery, SHR** | **Days at Home, IRR** |
| Unadjusted | 1.35 (1.10-1.66) | 0.93 (0.77-1.11) | 0.92 (0.75-1.13) |
| Adjusted using Claims Data | 1.23 (0.96-1.58) | 0.88 (0.70-1.18) | 0.97 (0.76-1.23) |
| Adjusted using Claims + EHR Data | 1.16 (0.90-1.52) | 1.04 (0.82-1.32) | 0.98 (0.76-1.27) |
| Abbreviation: LTACH, long-term acute care hospital; SNF, skilled nursing facility; HR, hazard ratio; SHR, subhazard ratio; IRR, incidence rate ratio  ^a^ Excluded 2,406 patients who met site-neutral payment criteria for reduced reimbursement (and thus less likely to be representative of the contemporary LTACH population) and 42 patients who received mechanical ventilation in either the LTACH or SNF (a population with less equipoise for LTACH transfer given less expertise and experience in SNFs). | | | |

| **Appendix Table S5.** Sensitivity Analysis for Healthcare Spending After LTACH vs. SNF Transfer among Hospitalized Adults Exempt from Site-Neutral Payment and Without Mechanical Ventilation (n=1,055) | | | | | |
| --- | --- | --- | --- | --- | --- |
|  | **LTAC, $** | **SNF, $** | **Difference, $ (95% CI)** | **P-value** |  |
| **Medicare Spending** |  |  |  |  |  |
| **Index episode of care^a, b^** |  |  |  |  |  |
| Unadjusted | 57,733 | 22,946 | 34,788 (30,679-38,896) | <.001 |  |
| Medicare model | 47,655 | 27,191 | 20,463 (17,681-23,245) | <.001 |  |
| Medicare & EHR model | 47,686 | 27,157 | 20,528 (17,659-23,398) | <.001 |  |
| **Post-Index episode of care^c,d^** |  |  |  |  |  |
| Unadjusted | 28,608 | 23,578 | 5,030 (269-9,792) | 0.04 |  |
| Medicare model | 22,265 | 26,709 | -4,444 (-9,207-319) | 0.07 |  |
| Medicare & EHR model | 22,699 | 26,470 | -3,770 (-8,810-1,269) | 0.14 |  |
| **Total 1-year spending^b^** |  |  |  |  |  |
| Unadjusted | 86,360 | 46,580 | 39,781 (32,637-46,924) | <.001 |  |
| Medicare model | 68,690 | 54,395 | 14,294 (8,177-20,411) | <.001 |  |
| Medicare & EHR model | 69,413 | 54,045 | 15,368 (8,873-21,862) | <.001 |  |
| **All Payer Spending** |  |  |  |  |  |
| **Index episode of care^a,b^** |  |  |  |  |  |
| Unadjusted | 61,084 | 26,141 | 34,943 (30,542-39,344) | <.001 |  |
| Medicare model | 50,695 | 30,628 | 20,067 (16,749-23,385) | <.001 |  |
| Medicare & EHR model | 45,959 | 26,546 | 19,414 (17,137-21,690) | <.001 |  |
| **Post-index episode of care^c,d^** |  |  |  |  |  |
| Unadjusted | 32,577 | 26,842 | 5,736 (328-11,143) | 0.04 |  |
| Medicare model | 25,326 | 30,409 | -5,082 (-10,490-326) | 0.07 |  |
| Medicare & EHR model | 25,676 | 30,235 | -4,559 (-10,251-1,132) | 0.12 |  |
| **Total 1-year spending^b^** |  |  |  |  |  |
| Unadjusted | 93,635 | 53,048 | 40,587 (32,848-48,326) | <.001 |  |
| Medicare model | 74,430 | 61,507 | 12,923 (6,216-19,631) | <.001 |  |
| Medicare & EHR model | 75,212 | 61,141 | 14,071 (6,948-21,194) | <.001 |  |
| Abbreviations: LTACH, long-term acute care hospital; SNF, skilled nursing facility  ^a^ Includes the initial acute care hospitalization and subsequent LTACH or SNF stay  ^b^ We computed marginal effects after gamma generalized linear regression model estimation with an identity link function to generate predicted spending for LTACH and SNF cohorts, and contrasts for the difference and robust standard errors using the delta method.  ^c^ Includes all hospitalizations and inpatient post-acute care stays (LTACH, SNF, IRF) after the index LTACH or SNF stay through 1-year after the date of transfer.  ^d^ We computed marginal effects after a two-part model, and contrasts for the difference and robust standard errors using the delta method. The first part of the model predicted whether spending for a patient was greater than zero. The second part, conditional on positive spending, predicted spending for LTACH and SNF patients using a gamma generalized linear regression model with a log link function. | | | | | |
